# Effectiveness of BNT162b2 COVID-19 Vaccine in Preventing Severe Symptomatic Infection among Healthcare Workers

**DOI:** 10.3390/medicina57080746

**Published:** 2021-07-23

**Authors:** Efrén Murillo-Zamora, Xóchitl Trujillo, Miguel Huerta, Mónica Riós-Silva, Oliver Mendoza-Cano

**Affiliations:** 1 Departamento de Epidemiología, Unidad de Medicina Familiar No. 19, Instituto Mexicano del Seguro Social, Av. Javier Mina 301, Col. Centro, Colima C.P. 28000, Mexico; efren.murilloza@imss.gob.mx; 2 Facultad de Medicina, Universidad de Colima, Av. Universidad 333, Col. Las Víboras, Colima C.P. 28040, Mexico; 3 Centro Universitario de Investigaciones Biomédicas, Universidad de Colima, Av. 25 de julio 965, Col. Villas San Sebastián, Colima C.P. 28045, Mexico; rosio@ucol.mx (X.T.); huertam@ucol.mx (M.H.); mrios@ucol.mx (M.R.-S.); 4 Facultad de Ingeniería Civil, Universidad de Colima, km. 9 Carretera Colima-Coquimatlán, Coquimatlán, Colima C.P. 28400, Mexico

**Keywords:** COVID-19 vaccines, BNT162 vaccine, COVID-19, SARS-COV-2

## Abstract

*Background and Objectives:* This study aims to evaluate the effectiveness of the BNT162b2 COVID-19 (coronavirus disease 2019) in preventing severe symptomatic laboratory-confirmed infection among healthcare workers in a real-world scenario. *Materials and Methods:* A cross-sectional analysis of a prospective cohort study was conducted. Subjects with onset illness from January to February 2021 were eligible and classified according to the number of vaccine doses received (single-shot, *n* = 8; two-shot, *n* = 12; unvaccinated, *n* = 290). *Results:* The vaccine effectiveness against severe illness was 100% in the single and two-shot group. The presented results suggest that vaccination reduces the frequency of severe symptomatic COVID-19 in working-age adults. *Conclusions:* Efforts focusing on maximizing the number of immunized subjects in the study population may reduce associated economic and social burdens.

## 1. Introduction

The burden of the coronavirus disease 2019 (COVID-19) caused by the severe acute coronavirus 2 (SARS-CoV-2) in Mexican healthcare workers has been high [1]. Factors determining this scenario include a high prevalence of comorbidities secondary to, among others, unhealthy dietary patterns and sedentary lifestyle in this population segment [2].

Passive immunization plays a critical role in reducing related morbidity and mortality. In Mexico, the BNT162b2 COVID-19 vaccine (Pfizer-BioNTech, New York Cit, NY, USA-Germany) was approved by federal authorities on 11 December 2020, and mass scale vaccination among first-line healthcare professionals started by the end of that month [3,4].

However, and to the best of our knowledge, there are no published studies evaluating the vaccination impact on this high-risk group and in the real-world. We aimed to evaluate the effectiveness of BNT162b2 COVID-19 vaccine in preventing severe symptomatic laboratory-confirmed infection in a subset of healthcare workers in a real-world scenario.

## 2. Materials and Methods

We performed a cross-sectional analysis of a prospective cohort study conducted in Mexico and on methods that had been broadly described [5]. Eligible subjects (aged 18 years and above) were laboratory-confirmed COVID-19 cases with illness onset from January to February 2021 and identified as healthcare workers (doctors, nurses, or other healthcare-related positions). 

Laboratory data included a positive reverse-transcription polymerase chain reaction (RT-PCR; Applied Biosystems, Waltham, MA, USA), or rapid antigen test result (Panbio COVID-19 IgG/IgM Rapid Test Device, Abbott Laboratories, Chicago, IL, USA; Standard Q COVID-19 Ag Test, SD Biosensor, Suwon, South Korea) in nasopharyngeal or deep nasal swabs. Severe disease was the main binary outcome, and it was defined by the presence of radiographic or clinical findings of pneumonia requiring hospital admission.

Participants were classified according to COVID-19 vaccination history as the following: unvaccinated (none shot or a single dose received at <14 days from acute illness onset), single-shot (one-shot received at 14 or more days from appearance of symptoms) and the two-dose group (adults with complete vaccination scheme). The employed cut-off (14 days) was based on the neutralizing antibodies response after the administration of the mRNA vaccine [6]. 

Summary statistics were computed, and the significance level was set at 5%. The Local Committee on Health Research (601) of the Mexican Institute of Social Security approved this study (R-2020-601-015).

## 3. Results

**Figure 1:**
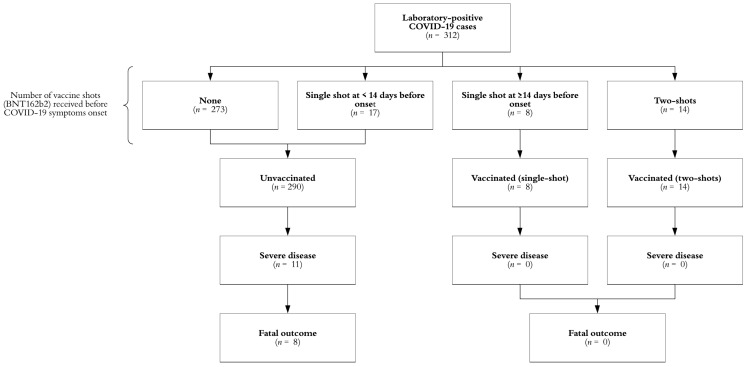
Study profile, Mexico 2021. Abbreviations: COVID-19, coronavirus disease 2019.

Data from 312 individuals were analyzed. The mean age (±standard deviation) of enrolled subjects was 39.3 ± 11.0 years old and most of them (59.3%) were female. The study profile is summarized in Figure 1.

Note: Scheme 19. was observed in 3.8% (*n* = 11/290) of unvaccinated subjects, most of them (*n* = 10) without a single vaccine dose received. The vaccine effectiveness against severe COVID-19 among subjects with a least a single shot was 100%. This scenario was observed, as shown in Table 1, despite that the vaccinated workers were older than the unexposed workers in general (*p* = 0.007).

No significant differences were observed among the analyzed groups in terms of the frequency of morbidities associated with the risk of a poorer disease outcome.

## 4. Discussion

Our findings suggest that vaccination efforts among Mexican healthcare workers had reduced the burden of COVID-19 in this high-risk group. Frontline healthcare professionals integrate the priority group immunized against the SARS-CoV-2 from December 2020 to February 2021 [7].

In addition to the prevalence of the imported variant 20C/S:452R (B.1.427/9, Epsilon, Irving, TX, USA) in Mexico, recent data revealed the circulation of three variants of interest in Mexico during the pre-vaccination stage. These variants show mutations in the spike protein, P.4 (B.1.1.28.4) and 20B/478K.V1 (B.1.1.222, leading to B.1.1.519), and in the nucleocapsid protein, 20A/N:194L.V2 (B.1.243) [8]. Further research is needed to determine the effectiveness of COVID-19 vaccines against these variants if they are still being transmitted during the post-vaccination stage.

The spread of the Delta variant (B.1.617.2) of SARS-CoV-2, which has already been already in 98 countries, poses a new challenge in the fight against COVID-19 [9]. By mid-July 2021, increasing rates in new COVID-19 cases had been documented in Mexico, coinciding with the relaxation of physical distancing and the spread of the Delta variant.

The limitations of our analysis must be cited. First, the sample size was small, and it was prohibitive to perform a stronger statistical analysis (i.e., evaluate factors associated with illness severity through risk ratios).

Second, we only included symptomatic cases of COVID-19, so the represented spectrum is partial. Third, we lacked data regarding the pre-existence of SARS-CoV-2 immunity, which plays a role in determining the analyzed infection [10]. Despite these limitations, our results are consistent with those previously published in the UK [11] and the USA [12].

## 5. Conclusions

These results suggest that vaccination reduces the frequency of severe symptomatic COVID-19 in working-age adults in a real-world scenario. Therefore, efforts focusing on maximizing the number of immunized subjects in the study population may reduce associated economic and social burdens.

## Figures and Tables

**Table 1:** Characteristics of the study sample for selected variables, Mexico 2021.

	Unvaccinated		BNT162b2 Vaccination Status				*p*
Characteristic			Single Dose		Two-Dose		
	*n* = 290		*n* = 8		*n* = 14		
Gender							
Female	171	(59.0)	6	(75.0)	8	(57.0)	0.651
Male	119	(41.0)	2	(25.0)	6	(43.0)	
Age (years) ^a^	39.0 ± 10.8		34.1 ± 10.2		47.5 ± 12.6		0.007
Diagnostic method							
RT-PCR	55	(19.0)	0	(0)	6	(42.9)	0.033
Rapid antigenic test	235	(81.0)	8	(100)	8	(57.1)	
COVID-19 severity							
Mild-moderate	279	(96.2)	8	(100)	14	(100)	0.649
Severe	11	(3.8)	0	(0)	0	(0)	
Outcome							
Recovery	282	(97.2)	8	(100)	14	(100)	0.732
Death	8	(2.8)	0	(0)	0	(0)	
Personal history of:							
Tobacco use (yes)	15	(5.2)	1	(12.5)	0	(0)	0.438
Obesity (yes)	41	(14.1)	1	(12.5)	2	(14.3)	0.991
Type 2 diabetes mellitus (yes)	17	(5.9)	0	(0)	2	(14.3)	0.707
Arterial hypertension (yes)	24	(8.3)	0	(0)	2	(14.3)	0.832
Chronic kidney disease (yes)	1	(0.3)	0	(0)	0	(0)	0.961

Abbreviations: RT-PCR, reverse-transcription polymerase chain reaction; COVID-19, coronavirus disease 2019. Notes: (1) The absolute (*n*) and relative (%) frequencies are presented except if the mean is specified; (2) unvaccinated subjects were those with any vaccine shot before onset of COVID-19 symptoms or a single dose at <14 days before appearance of symptoms; (3) *p*-value from chi-squared or ANOVA test, as corresponding; (4) severe disease was the main binary outcome, and was defined by the presence of radiographic or clinical findings of pneumonia requiring hospital admission. ^a^ Arithmetic mean ± standard deviation.

## Data Availability

The datasets generated during and/or analyzed during the current study are available from the corresponding author on reasonable request.
